# The Zahn drawings: new illustrations of *Xenopus* embryo and tadpole stages for studies of craniofacial development

**DOI:** 10.1242/dev.151308

**Published:** 2017-08-01

**Authors:** Natalya Zahn, Michael Levin, Dany Spencer Adams

**Affiliations:** 1 www.natalya.com, Cambridge, MA, USA; 2 Department of Biology and Tufts Center for Regenerative and Developmental Biology, Tufts University, Medford, MA 02155, USA; 3 Allen Discovery Center at Tufts University, Medford, MA 02155, USA

**Keywords:** *Xenopus laevis*, Embryonic development, Stage series, Craniofacial patterning

## Abstract

The embryos and tadpoles of the frog *Xenopus* are increasingly important subjects for studies of the development of the head and face – studies that are providing novel and crucial insight into the causes and prevention of a suite of devastating birth defects, as well as basic evolutionary and developmental biology. However, many studies are conducted on a range of embryonic stages that are not fully represented in the beloved *Xenopus* resource, Nieuwkoop and Faber's classic *Normal Table of* Xenopus laevis *(Daudin)*. The lack of standardized images at these stages acts as a barrier to the efficient and accurate representation and communication of experimental methodology and expression data. To fill this gap, we have created 27 new high-quality illustrations. Like their oft-used predecessors from Nieuwkoop and Faber, these drawings can be freely downloaded and used, and will, we hope, serve as an essential resource for this important model system.

## Abstract (precis)

**Summary:** New, freely available illustrations of developing *Xenopus laevis*, drawn by Natalya Zahn, provide a resource for teaching and research, especially in the field of craniofacial biology.

## Introduction

**Fig. 1.**
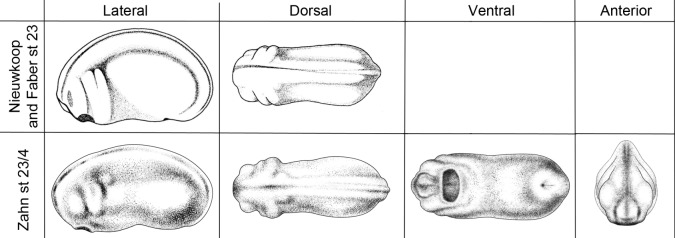
**Comparison of the styles of the drawings by Prijs and Zahn.** Lateral and dorsal drawings of stage 23 *Xenopus* from Nieuwkoop and Faber (1994) and of stage 23/24 *Xenopus* by Natalya Zahn for comparison of the styles. Drawings of stage 24 *Xenopus* ventral and anterior views by Zahn are also shown. The goal was for the styles to be similar enough for the Zahn drawings to be put to use as quickly as possible, yet distinct enough for the correct artist to be recognized and acknowledged. Drawings by Natalya Zahn licensed under CC-BY-NC at http://ase.tufts.edu/biology/faculty/adams/zahnDrawings.htm and reproduced with permission.

In 1956, the editors Peter Nieuwkoop and Job Faber published the *Normal Table of* Xenopus laevis *(Daudin)* (hereafter referred to as Normal Table), a work whose practical importance to developmental biology cannot be overstated. Part of that volume, perhaps the most frequently used part, is a set of drawings by J. J. Prijs that were based on pencil drawings by Job Faber (Nieuwkoop and Faber, 1956). These different views of *Xenopus* development illustrate the changing appearance of the embryo as it matures from egg to adult frog. These drawings, like those of zebrafish (Kimmel et al., 1995) (https://zfin.org/zf_info/zfbook/stages/figs/fig1.html) have become indispensable tools not only for teaching and identifying important differences between stages, as well as changes due to manipulation, but also for reporting results and interpretations. The *Xenopus laevis* embryo is a valuable model for molecular and cell biological, biophysical and physiological approaches to medicine, development, immunology, birth defects research, cardiac and kidney disorders, neuroscience, genetics, cancer, drug screening, regeneration and evolutionary biology (Abu-Daya et al., 2012; Adams, 2008; Adams et al., 2014; Cross and Powers, 2009; Duncan and Khokha, 2016; Goyos and Robert, 2009; Hardwick and Philpott, 2015; Heikkila et al., 2007; Henry et al., 2008; Kaltenbrun et al., 2011; Kinney and Cohen, 2009; Koide et al., 2005; Lee-Liu et al., 2016; Levin, 2013; Pegoraro and Monsoro-Burq, 2013; Pratt and Khakhalin, 2013; Robert and Cohen, 2011; Robert and Ohta, 2009; Schmitt et al., 2014; Slack et al., 2008; Straka and Simmers, 2012; Suzuki et al., 2006; Takagi et al., 2013; Tseng and Levin, 2013; Vandenberg et al., 2013; Warkman and Krieg, 2007; Wheeler and Liu, 2012). The availability of a standard template onto which researchers can, and frequently do, project their novel findings has led to clarity in communication, despite the challenges inherent in describing patterns and shapes that are three dimensional and, by definition, constantly changing. Two new editions, one in 1967 and another in 1994, attest to the continued importance of this resource; all of the drawings from the book are already available on Xenbase: (http://www.xenbase.org/anatomy/alldev.do).

Among the above-referenced fields of study, the embryos and tadpoles of *Xenopus* are increasingly important subjects for studies of the development of the head and face, studies that are providing novel and crucial insight into the causes and prevention of a suite of devastating birth defects. To educate the next generation of researchers, to prevent unnecessary duplication of results, and to accurately communicate the results of these studies and thus build up a foundation of shared knowledge, it is crucial to have a centralized resource comprising standard descriptions and illustrations of normal anatomy and physiology (Jacox et al., 2014; Kennedy and Dickinson, 2014; Slater et al., 2009). The frequently used drawings in Nieuwkoop and Faber's Normal Table, however, lack many stages and views of importance to the growing community of scientists studying craniofacial development. We therefore commissioned the natural science illustrator Natalya Zahn to create the illustrations described here.

## Planning the illustrations

After many helpful communications with interested scientists, we decided on our approach. Two criteria were used to decide which stages and views would be included. First, the view would not be available in the Normal Table. The only exceptions are a lateral and a dorsal view of a stage 23 embryo that we show side by side with Prijs' illustrations (Fig. 1) to allow users to compare the drawing styles. Second, the drawing would be useful for illustrating important changes during craniofacial development. To fulfill this requirement, we consulted the text of the Normal Table (1994 edition, Nieuwkoop and Faber, 1994) for information about the concurrent status of internally developing organs, such as the optic lobe, and we made our own observations of the changing appearance of the embryo and tadpole stages.

Importantly, we wanted to keep the drawing style similar enough to Prijs' to allow easy comparison and immediate use of these new drawings in laboratories and publications. However, it was also essential that the style should be distinct enough to avoid confusion concerning attribution. Moreover, we set out to make these illustrations available to researchers via the same routes and under the same rules as Prijs' drawings, i.e. freely available on Xenbase (James-Zorn et al., 2015) with the appropriate citation.

## Producing the illustrations

The process for the creation of all new illustrations began with observation of live specimens under a Nikon SMZ1500 stereomicroscope. Animals used for this project were maintained by D.S.A. according to approved IACUC protocol M2014-79 and IRB protocol MR-58 from Tufts University (MA, USA). D.S.A. raised and staged animals, chose representative individuals, and performed all manipulating, orienting and euthanizing of embryos and tadpoles. Embryos were held at different orientations using wells made in agar dishes; individuals with active musculature were anesthetized using 0.1% MS-222 (tricaine).

Embryos were staged according to Nieuwkoop and Faber (1994), and multiple samples of each developmental stage were reviewed to account for individual idiosyncrasy, thereby allowing the artist to create an ‘average’ animal; this was especially important for stages with highly variable pigmentation. Sketches of these live samples were made in the lab by Zahn, noting any distinct embryo features; each sample and view was captured using a digital micrograph for later reference and for posting online with the finished illustrations.

To take advantage of the efficiency and ease of continuous editing, all final illustrations were executed digitally using applications from Adobe's Creative Suite and a Wacom Intuous 4 digital drawing tablet. Using lab sketches and associated photographs as guides, primary outlines and major feature landmarks for each embryo stage and/or view were drawn in vector format using Adobe Illustrator; vector lines were assigned custom stroke effects, lending an organic, ink-drawn feel. Drawings were then imported into Adobe Photoshop and shaded using specialized digital brushes that mimic pen and ink stippling. The end result is a collection of illustrations that share the same general character and classic utility as the Nieuwkoop and Faber drawings, with a slightly cleaner and more modernized polish.

**Fig. 2.**
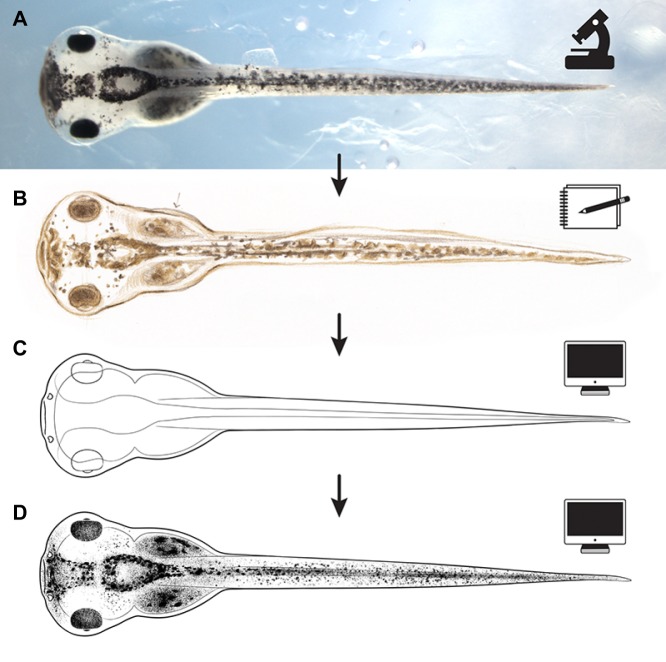
**Evolution from the view through the microscope to the final digital image.** Anterior is towards the left in all images. (A) The micrograph of one of the *Xenopus* embryos used by Zahn to draw preliminary sketches of the dorsal view of a stage 43 tadpole. (B) Zahn's preliminary sketch of a stage 43 tadpole, dorsal view, based on visual examination of two or three examples, including that shown in A. (C) Vector drawing made in Adobe Illustrator based on the sketch and the micrographs. (D) Final drawings include shading added using Adobe Photoshop.

**Fig. 3.**
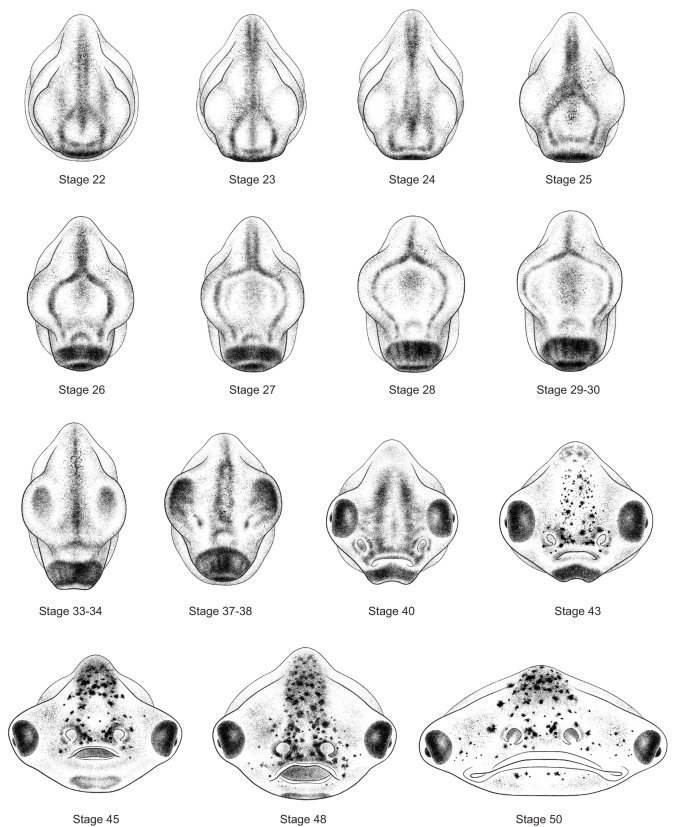
**Anterior views of 15 stages of *Xenopus* development.** Stage 48 is shown with mouth open. Drawings by Natalya Zahn licensed under CC-BY-NC at http://ase.tufts.edu/biology/faculty/adams/zahnDrawings.htm and reproduced with permission.

**Fig. 4.**
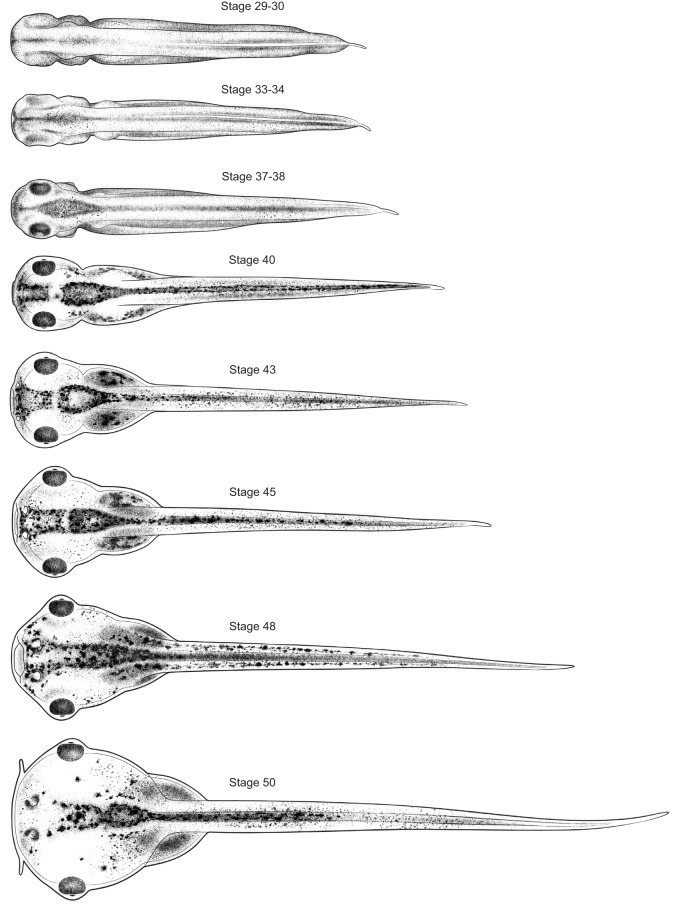
**Dorsal views of 8 stages of *Xenopus* development.** Anterior is towards the left. Stage 48 is shown with mouth open. Drawings by Natalya Zahn licensed under CC-BY-NC at http://ase.tufts.edu/biology/faculty/adams/zahnDrawings.htm and reproduced with permission.

**Fig. 5.**
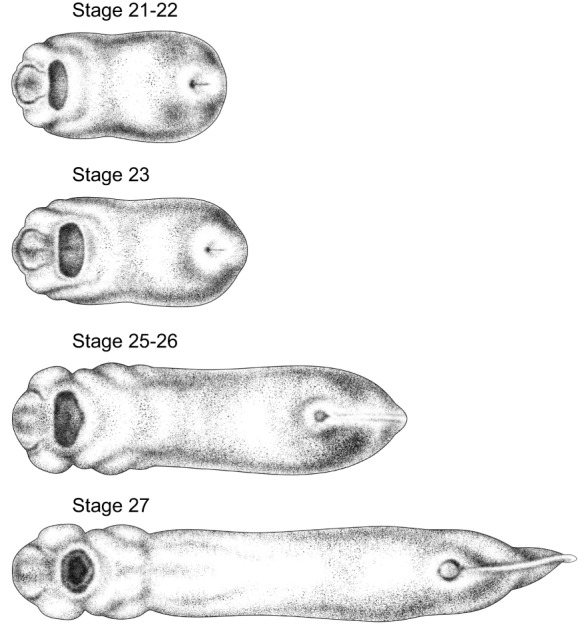
**Ventral views of 4 stages of *Xenopus* development.** Anterior is towards the left. Drawings by Natalya Zahn licensed under CC-BY-NC at http://ase.tufts.edu/biology/faculty/adams/zahnDrawings.htm and reproduced with permission.

To illustrate the process used by Zahn to create her drawings, Fig. 2 shows the evolution of one drawing from view through the microscope to finished product. Figs 3-5 show the complete set of drawings, arranged by orientation and stage.

## Making the Zahn drawings available to the community

The Zahn drawings are available at http://ase.tufts.edu/biology/faculty/adams/zahnDrawings.htm and will be hosted permanently at http://www.xenbase.org as part of XenHead, an atlas comprising brightfield images, physiology, and other types of data of interest to those studying craniofacial development. Additional illustrations by Zahn, including simple line drawings useful for illustrating expression patterns, will be added as they become available. Here, we introduce the final drawings grouped by angle of view. In this format, the changes over time are apparent as never before.

## Concluding remarks

Because the Zahn drawings show views that have not been illustrated this way before, they draw attention to as yet unstudied morphological changes during crucial stages of organogenesis. Particularly noticeable is the change in both the shape and size of the head. Although sufficient for staging, Prijs' profiles of these same stages give no hint of the radical changes taking place. Thus, beyond providing standardized drawings onto which we can project our findings, these new drawings also reveal new avenues of inquiry.

Another difference between these drawings and Prijs' are that these were drawn from life, by the artist, from multiple examples. Prijs was working from sketches made by Faber, and thus was not able to adjust for biological variation, nor was he able to vary focus to confirm his interpretations of what he was seeing. The difference is especially clear in Fig. 1, which compares two drawings of the same stage. In fact, we chose this stage for Zahn to re-draw specifically for this comparison because of the discrepancies we had already noted between Prijs' drawings and our impression of embryos at this stage. Although there are no other drawings of equivalent stages and views, we are confident that these new drawings are as accurate as, or in some cases more accurate than, the drawings with which we all have worked to date.

Our hope is that the Zahn drawings will prove as useful as Prijs' classic illustrations. In addition, we encourage other researchers to commission additional drawings of different *Xenopus* views or stages (and of other species where current reference illustrations are incomplete). To do so, scientists should contact Natalya Zahn directly; however, we would invite anyone commissioning new *Xenopus* images to consider allowing them to be hosted at Xenbase, along with those presented here, to be shared freely with the community at large. All of the drawings, published at Xenbase under a CC-BY-NC license, are freely available for non-commercial use. Members of the community are welcome to reproduce and use these drawings with appropriate attribution to the artist and this article.
